# Computational Study of the Dimerization of the Parathyroid Hormone

**DOI:** 10.1021/acs.jpcb.5c06649

**Published:** 2025-11-21

**Authors:** C. Sommerfeld, W. Paul

**Affiliations:** Department of Physics, Martin-Luther-University Halle-Wittenberg, 06099 Halle, Germany

## Abstract

We present computer simulations of the dimerization process of three different parathyroid horomone (PTH) segments, PTH_34_, PTH_42_, and PTH_84_. A thermodynamic analysis reveals that the aggregation is driven and determined by the N-terminal segment PTH_34_ alone. Our simulation model is a coarse-grained one but we can translate the temperature scale of our simulation to physical units by comparison of chain size with experimental data. From this, we can identify that the dimerization and concomitant chain folding occurs only slightly above physiological temperature conditions. The noncooperativity of the dimerization process and its vicinity to physiological conditions give rise to the reversibility of the aggregation of PTH and the functional use of PTH fibrils as storage devices.

## Graphical abstract

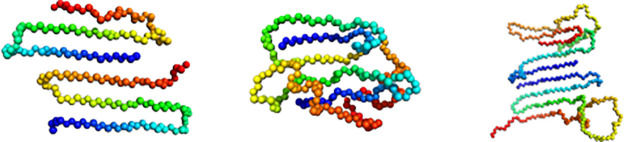

## Introduction

The formation of amyloid fibrils is mostly studied in the context of neuro-degenerative deseases, like Alzheimer’s, Parkinson’s, or Huntington’s desease.^1^ It is, however, a quite general aggregation phenomenon of proteins in supersaturated solutions and can even serve functional purposes as a protein storage device. This has been observed for secretory proteins, among them calcitonin.^2^ Calcitonin and its antagonist, the parathyroid hormone (PTH), are secretory proteins regulating the calcium and phosphate content in our blood by changing uptake or release of these in the bones and kidneys. PTH readily forms amyloid fibrils in vitro^3–5^ but stays monomeric in vivo, where amyloid formation can be induced by the presence of heparin.^6^ Being on the edge of fibrillation under physiological conditions, it is hypothesized that PTH shares the trait of calcitonin to be able to form functional fibrils, serving the purpose of storage device when lower PTH content in the blood is needed for calcium regulation purposes.

In its physiological form, PTH consists of 84 amino acids (PTH_84_). In vitro, it possesses the special property that it has a very high critical solution concentration of *c*_crit_ = 79 ± 4 μM,^4^ so the monomeric state (single protein state) and the equilibria between monomers, dimers, trimers, tetramers, and the mature fibril could be studied in detail.^4,5^ At low concentration, one observes at room temperature a chemical equilibrium between monomer and dimer and close to the critical concentration trimers and tetramers start to form, before the mature fibril nucleates.

The fibril formation,^3^ same as the dimerization,^4^ is driven by hydrogen bonding interactions of the first 34 N-terminal amino acids (PTH_34_) with some contribution from amino acids in the center of the PTH_84_ chain. Both in the complete protein^4^ and the N-terminal segment, the secondary structure of the monomer in solution consists of two α-helical segments in the N-terminal part (PTH_34_) joined by a flexible linker,^7–12^ the rest of the chain is unstructured. This could also be confirmed by Monte Carlo simulations.^13^ Upon dimerization and fibril formation, the protein, therefore, has to undergo a structural change from α-helical to β-sheet behavior.

The structure of single PTH_34_ chains in solution has also been studied for an all-atom protein model using flat-histogram Monte Carlo simulations, in the one case, a multicanonical simulation,^14^ including protein–solvent interactions in the form of an energy modeling solvent accessible surface, in the other case, a Wang–Landau simulation,^15^ using only the all-atom force field for the protein. The first one performed a thermodynamic and secondary structure analysis of the conformations of PTH_34_ over a broad temperature range; the second one only reported on a thermodynamic analysis. In the simulation employing the solvent accessible surface energy, a single transformation from a high-temperature random coil structure to a low-temperature α-helical state occurred at about 560 K. In the simulation modeling only the protein, the same transition peak in the specific heat was found, but in addition, a second transition occurred at a lower temperature of 424 K. The high temperature peak here signified a collapse of the chain as measured by the radius of gyration as a function of temperature, and the authors argued, but did not show, that the low temperature signified the transition to the α-helical state. Hansmann^14^ had already concluded that his simulation model severely overstabilizes the helical state compared to experimental findings, so a much lower folding temperature seems more realistic.

We performed a comparative simulation study of the thermodynamics of dimerization of PTH segments of three different lengths, the physiological one PTH_84_, the N-terminal segment often studied for its aggregation behavior, PTH_34_, and an intermediate length one, PTH_42_, including chain parts which showed hydrophobic aggregation propensities in experiments.^4^ We used an intermediate resolution protein model, PRIME20,^16^ which had been optimized against the protein data bank. To be able to quantify the thermodynamic behavior over a broad temperature range, we employ Stochastic Approximation Monte Carlo (SAMC),^17,18^ a flat-histogram Monte Carlo method.

## Methods

To investigate the thermodynamic properties and conformational ensembles of human parathyroid hormone (PTH) dimers of varying chain lengths (PTH_34_, PTH_42_, and PTH_84_), we employed the PRIME20 coarse-grained protein model in conjunction with the Stochastic Approximation Monte Carlo (SAMC) sampling algorithm. This methodological combination provides efficient access to the dimerization behavior and structural transitions of PTH variants across a comprehensive temperature range.

### PRIME20 Model

The PRIME20 model is a coarse-grained protein representation of intermediate resolution, first introduced by Cheon et al.^16^ as an extension of the PRIME approach (**PR**otein **I**nter**ME**diate resolution). PRIME20 incorporates parametrizations for all 20 standard amino acids and has demonstrated broad applicability for studying protein folding, association, and aggregate formation.^16,19–22^

In PRIME20, each amino acid is mapped onto four interaction sites: three backbone beads representing the amine nitrogen (NH), the Cα carbon, and the carboxyl carbon (CO), as well as a side-chain bead positioned at the side chain’s center of mass. The side-chain beads capture amino-acid-specific size, position, and interaction properties. Detailed parameter values are provided in ref 21 and the doctoral dissertation of Böker.^23^ The left figure in Figure 1 illustrates this bead mapping, using color codes for backbone and side-chain groups.

**Figure 1:**
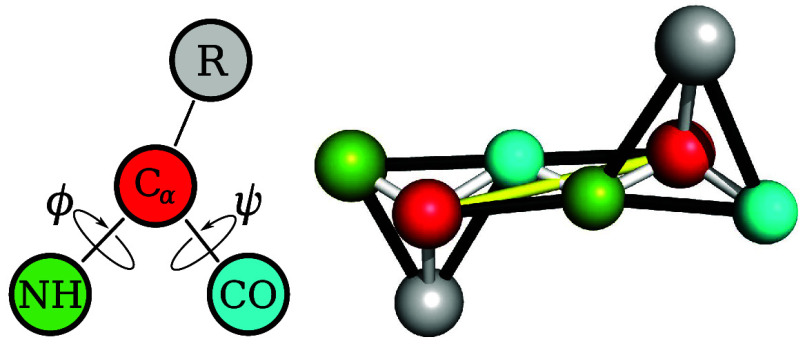
Coarse-grained representation of amino acids in PRIME20. Left: bead mapping; right: bond and pseudobond configuration. Covalent bonds are white, pseudobonds enforcing bond angles are black, and the yellow bond enforces the trans configuration of the peptide bond.

The backbone’s three-bead construction enables explicit calculations of the peptide dihedral angles ϕ and ψ, facilitating the geometrically resolved modeling of hydrogen bonding patterns relevant to secondary structure formation.

Covalent connections are encoded via square-well potentials centered on ideal bond lengths *r*_ideal_, with a tolerance parameter Δ = 0.02375

1 $$V_{\mathrm{bond}}\left(r_{\mathrm{ij}}\right)=\{\begin{array}{ll} 0 & r_{\mathrm{ideal}}\left(1-\Delta \right)\leq r_{\mathrm{ij}}\leq r_{\mathrm{ideal}}\left(1+\Delta \right)\\ \infty & \mathrm{otherwise} \end{array}$$

Here, *r*_*ij*_ is the bead–bead distance. The right figure in Figure 1 shows both covalent (white) and pseudobonds (black and yellow), the latter enforcing key backbone geometries, such as the peptide bond’s *trans* configuration (yellow). All relevant bond lengths and angles are summarized in Table 1. Residue-specific details can be found in ref 21.

**Table 1:** Characteristic Bond and Pseudobond Lengths, Bead Diameters, and Backbone Angles Used in the PRIME20 Model^a^

*b*_*ij*_		*b*_*ij*_^(pseudo)^	
NH–Cα	1.46	NH*_i_*–CO_*i*+1_	4.25
Cα–CO	1.51	Cα*_i_*–NH_*i*+1_	2.41
CO–NH	1.33	CO*_i_*–Cα_*i*+1_	2.45
		Cα_*i*_–Cα_*i*+1_	2.80

*d*_*ij*_^(HS)^		bond angle	
NH	3.3	∠(NH–Cα–CO)	1.937
Cα	3.7	∠(Cα–CO–NH)	2.025
CO	4.0	∠(CO–NH–Cα)	2.129

a All values are given in Å or radians, as indicated.

Noncovalent interactions are implemented using hard-sphere and square-well potentials. Bead overlap is prevented by hard-core repulsion, with bead diameters *d*_*i*_^(HS)^ combined via the Lorentz–Berthelot rule

2 $$d_{\mathrm{ij}}^{\left(\mathrm{HS}\right)}=\frac{1}{2}\left(d_{i}^{\left(\mathrm{HS}\right)}+d_{j}^{\left(\mathrm{HS}\right)}\right)$$

Side chain–side chain and hydrogen bond interactions are captured via square-well potentials

3 $$V_{\mathrm{ss}}\left(r_{\mathrm{ij}}\right)=\{\begin{array}{ll} \infty & \mathrm{if}\;0\leq r_{\mathrm{ij}}\leq d_{\mathrm{ij}}^{\left(\mathrm{HS}\right)}\\ \varepsilon_{\mathrm{ij}} & \mathrm{if}\;d_{\mathrm{ij}}^{\left(\mathrm{HS}\right)}\leq r_{\mathrm{ij}}<d_{\mathrm{ij}}^{\left(\mathrm{SW}\right)}\\ 0 & \mathrm{if}\;d_{\mathrm{ij}}^{\left(\mathrm{SW}\right)}\leq r_{\mathrm{ij}} \end{array}$$

Here, *d*_*ij*_^(SW)^ sets the interaction range, and ε^*ij*^, the depth of the well. For backbone hydrogen bonds, *d*_*ij*_^(SW)^ = 4.5 Å, with an interaction energy of *E*_HB_ = −1.0, which defines the model’s energy scale. Reduced units are used throughout: all energies are given in multiples of ε_HB_, and temperatures in units of ε_HB_/*k*_B_. For conversion to physical units, absolute values of ε_HB_ must be determined as described in ref 21. The model parametrization reflects equilibrium conditions analogous to physiological environments, and, as with any coarse-grained approach, quantitative accuracy may decrease away from those regimes.

Geometric constraints on hydrogen bonding are modeled by requiring acceptable relative positioning between virtual hydrogen bond donors and acceptors, computed from backbone bead coordinates.^21,23^

For side chain to backbone contacts, the side chain’s self-interaction-diameter is used. To allow for realistic chain flexibility and avoid steric constraints that can arise due to coarse mapping, the model employs so-called *squeeze parameters*. These adjust effective bead diameters where necessary to permit sampling of backbone and side-chain conformations that would otherwise be sterically forbidden.^21,23^ Table 2 lists the interactions where these corrections are applied.

**Table 2:** Bead Pairings Affected by Squeeze Parameters in PRIME20^a^

BB–BB	SC–BB
Cα_*i*_–CO_*i*+1_	Cα_*i*–1_–R_*i*_
Cα_*i*_–NH_*i*+2_	CO_*i*–1_–R_*i*_
CO_*i*_–NH_*i*+2_	NH_*i*+1_–R_*i*_
NH_*i*_–NH_*i*+1_	Cα_*i*+1_–R_*i*_
CO_*i*_–CO_*i*+1_	CO_*i*–2_–R_*i*_

a BB–BB: Backbone–backbone. SC–BB: Side chain–backbone. The squeeze modifies the effective excluded volume for specific pairs to enable realistic conformational sampling.

### Stochastic Approximation Monte Carlo

The conformational landscape of PTH dimer systems was explored using the Stochastic Approximation Monte Carlo (SAMC) algorithm.^17^ SAMC is well-suited for sampling systems with complex energy landscapes. It is part of the broader class of flat-histogram methods, which efficiently traverse regions of both high and low probability by aiming for uniform sampling over energy states.

The central quantity in SAMC is the density of states (DOS), *g*(*U*), as a function of the system’s potential energy *U*. Since *g*(*U*) is unknown beforehand, the algorithm employs a recursively updated estimate *g̃*(*U*), starting from an initial uniform guess. Monte Carlo moves are proposed from configuration *x* (energy *U*(*x*)) to *x*′ (energy *U*(*x*′)), and accepted with probability

4 $$w\left(x\to x^{\prime }\right)=\min\left(1,\frac{\mathrm{g̃}\left(U\left(x\right)\right)}{\mathrm{g̃}\left(U\left(x^{\prime }\right)\right)}\right)$$

After each move, the DOS estimate is refined

5 $$\ln{\mathrm{g̃}}_{t+1}\left(U\left(x_{\mathrm{new}}\right)\right)=\ln{\mathrm{g̃}}_{t}\left(U\left(x_{\mathrm{new}}\right)\right)+\gamma_{t}$$

where γ_*t*_ is a modification factor that decreases with the simulation time *t*. This controlled reduction allows convergence to an accurate DOS, as outlined in refs 18,24,25 .

Because *g*(*U*) spans many orders of magnitude, the logarithm ln *g*(*U*) is used in practice, which conveniently corresponds to the microcanonical entropy *S*(*U*). Throughout this study, *S*(*U*) was averaged over eight independent simulation replicas for statistical reliability. The uniformity of the visited energy histogram, *H*(*U*), serves as an indicator of sampling quality.

Upon achieving a stable DOS estimate, we performed production simulations using a fixed *g̃*(*U*) to collect statistics on structural observables relevant for PTH dimers, such as the gyration tensor $T_{g}\left(U\right)$ and hydrogen bond counts *N*_HB_(*U*), as function of potential energy.

The MC move set consists of (i) local random displacements of beads within 0.2 Å, (ii) pivot rotations modifying ϕ or ψ dihedrals by up to π/3, and (iii) rigid-body translation or rotation of entire chains, enabling comprehensive sampling of intra- and interchain configurations. All proposed moves are checked for steric compatibility, and moves resulting in bead overlaps are rejected.

The simulations were conducted in a cubic box of linear dimension *L* = 300 Å, corresponding to peptide (monomer) concentrations of *c* ≈ 100 μM, consistent with experimentally accessible in vitro conditions. Experimentally, the aggregation behavior of PTH was studied over a wide concentration change ranging from a few to a few hundred μM and a critical concentration of 79 ± 4 μM^4^ for the formation of fibrils was found.

## Results and Discussion

The SAMC method iteratively determines the density of state *g*(*U*) of a model as a function of its potential energy. From this function, one can obtain the true microcanonical density of states *g*(*E*) as a function of the total energy *E*, including the kinetic energy, by convoluting the configurational density of states with the kinetic density of states. The latter is equal to the density of states of an ideal gas.^26,27^ In Figure 2, we are comparing the logarithms of the densities of state, *S*(*E*) = ln­(*g*(*E*)), i.e., their microcanonical entropies, for all three chain lengths.

**Figure 2:**
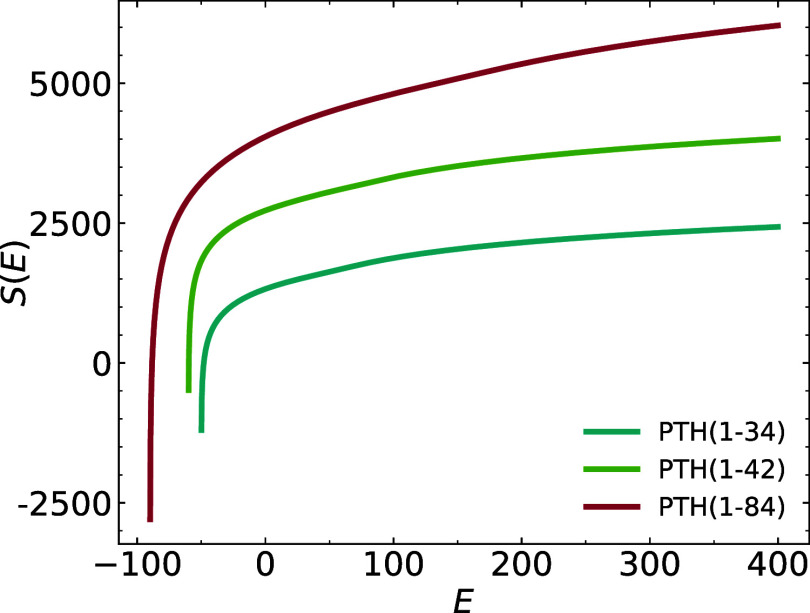
Microcanonical entropy as a function of total energy for the three PTH chain lengths in comparison (curves are shifted vertically for clarity).

The high energy behavior of this function is given by the ideal gas limit, the low energy limit is set by the lowest potential energy found in the simulation. All thermodynamic information about our model system is contained in these functions. A microcanonical analysis uses derivatives of the entropy with respect to energy; a canonical analysis transforms to the canonical partition function first

6 $$Z\left(T\right)=\sum_{E}g\left(E\right)\;\exp\left\{-\beta E\right\}$$

and determines the thermodynamics from there. For the small systems we are studying, both approaches are not equivalent like in the thermodynamic limit, but can offer complementary information.

### Thermodynamics of Dimerization

In the microcanonical picture, the temperature can be calculated through *T* ^–1^(*E*) = ∂*S*(*E*)/∂*E* and the specific heat is given as *C*(*E*) = 1/*T* ^2^(*E*) ∂*T* ^–1^(*E*)/∂*E*. In the canonical picture, one has *C*(*T*) = ∂⟨*E*⟩(*T*)/∂*T* and ⟨*E*⟩(*T*) = 1/*Z*(*T*)∑_*E*_ *Eg*(*E*) exp­{−β*E*}.

In Figure 3, we are showing on the left side the microcanonical temperature and specific heat, and on the right side, the canonical specific heat for all three chain lengths. In the microcanonical temperature, a first-order phase transition shows up as a loop region, a second order phase transition as an isolated inflection point. The loop behavior translates into singularities in the specific heat bordering one (or more) maxima with values below zero. The isolated inflection point translates into a specific heat maximum with value larger than zero. Our finite (small) systems, of course, do not possess real phase transitions like in the thermodynamic limit, but the typical signature of such transitions is already well established, so that we will use the terms first or second order phase transitions in the following. The two peaks within the first-order loop on the left can no longer be resolved in the canonical specific heat on the right but give rise to one high temperature first-order transition peak. The low energy second order maximum in the microcanonical analysis gives rise to the second maximum in the canonical analysis at lower temperature, which for PTH_34_ is only a shoulder on the specific heat curve. Our findings, therefore, are better in accord with the result of Hansmann that there is only one transition peak for PTH_34_ at which collapse and secondary structure formation can be expected to occur simultaneously. Physically, this is reasonable as both, collapse and ordering, are driven by the hydrogen bonding interaction. In our case, this energy scale sets the scale for the dimerization temperature as well, so we expect—and will show in the following—that aggregation, collapse, and secondary structure formation occur simultaneously. For the two longer chain segments there is, however, a clear lower temperature phase transition peak and we will clarify its significance. The transition temperatures in the microcanonical and canonical ensemble are listed in Table 3. The first-order transition temperatures in the microcanonical ensemble are given by the Maxwell-like loop construction on Figure 3. Where resolvable, transition temperatures in the two ensembles agree well with each other.

**Figure 3:**
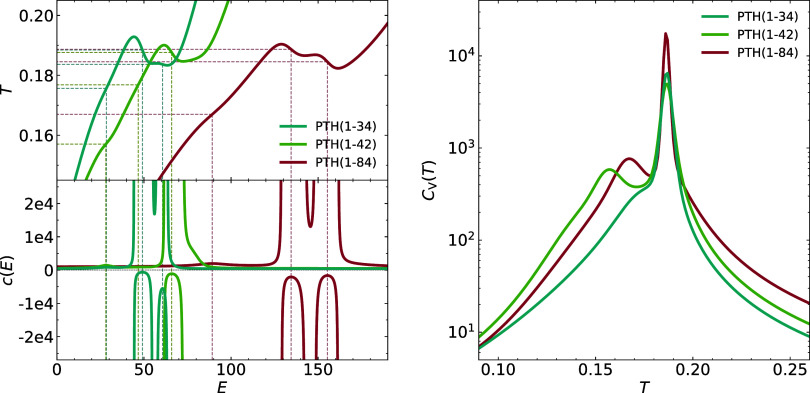
Left plot shows the microcanonical temperature and specific heat, and the right figure shows the canonical specific heat. Both plots show data for all three chain lengths investigated.

**Table 3:** Phase Transition Temperatures T* from Microcanonical and Canonical Analysis

system	*T*_first_^*micro^	*T*_second_^*micro^	*T*_high_^*can^	*T*_low_^*can^
PTH_34_	0.1867	0.1756	0.187	
PTH_42_	0.1867	0.157	0.187	0.157
PTH_82_	0.1864	0.167	0.186	0.167

A special feature of the three canonical specific heats shown on the right side of Figure 3 is the fact that the aggregation/ordering peak is at the same temperature for all three chain lengths. This is an unusual behavior as, typically, transition temperatures increase with increasing system size. It is a clear indication that the aggregation and ordering in these protein chains is not a phenomenon that is collective with respect to the whole chain, but only with respect to the short N-terminal PTH_34_ segment. This segment is responsible for the aggregation and undergoing secondary structure formation, the rest of the chain is not involved in the main transition. That part is responsible for the lower temperature specific heat peak which is absent for the short segment, but already present for the slightly longer PTH_42_ segment.

In Figure 4, we are showing the number of hydrogen bonds formed within a chain (secondary structure formation) and between the two chains (dimerization). The number of intermolecular hydrogen bonds formed in the N-terminal segment PTH_34_ is clearly almost equal to the number of such bonds formed in the two longer segments. From this, we can conclude—in agreement with the literature—that this part of the chain drives the aggregation into the dimer (and consecutively the fibril) and that it is also this part of the chain that undergoes a secondary structure formation at this transition temperature. We will analyze the structural properties of the dimer in the next section.

**Figure 4:**
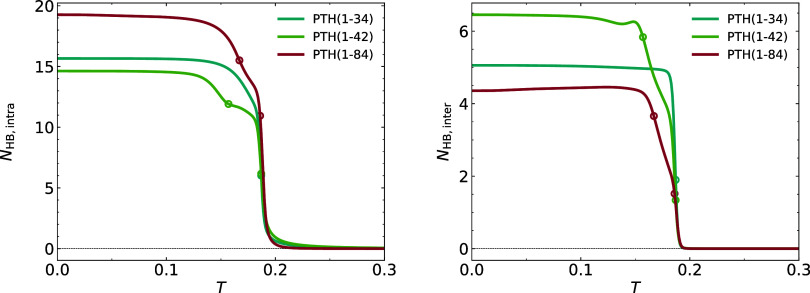
Left plot shows the number of intramolecular hydrogen bonds as a function of temperature, the right plot shows the number of intermolecular ones. The open circles show the transition temperatures obtained from the specific heat.

### Structural Properties of the Dimers

To sample structural properties as a function of energy, we use the converged density of states shown in Figure 2 for a long simulation visiting all possible energy states of the system. For all energy values, we determine structural quantities like the radius of gyration of the chain (or also the number of hydrogen bonds shown in Figure 4). Let *f*(*E*) denote a structural quantity then

7 $$\mathrm{f̅}\left(E\right)=\frac{1}{M\left(E\right)}\sum_{n=1}^{M\left(E\right)}f_{n}\left(E\right)$$

where *M*(*E*) is the number of visits to energy *E*, is the microcanonical expectation value of quantity *f* at energy *E*. From this one can obtain its temperature dependence by

8 $$f\left(T\right)=\frac{1}{Z\left(T\right)}\sum_{E}\mathrm{f̅}\left(E\right)g\left(E\right)\;\exp\left\{-\beta E\right\}$$

One important such quantity is the well-known radius of gyration, which we can determine for the monomer as well as the dimer of, e.g., PTH_84_. Another quantity measuring the shape of the chains is the parameter

9 $$\kappa^{2}=\frac{\lambda_{1}-\lambda_{3}}{\lambda_{1}+\lambda_{2}+\lambda_{3}}$$

where the λ_*i*_ are the eigenvalues of the gyration tensor of a chain. A value of κ^2^ ∼ 0.5 is typical of a random coil state, κ^2^ = 0 indicates a spherical shape, and κ^2^ = 1 indicates a rod-like object.

In Figure 5, we show on the left side the radius of gyration of a PTH_84_ monomer as well as its dimer. These quantities had been determined experimentally.^4^ We use a comparison with these experiments to perform a temperature gauge for our simulations, i.e., to determine the average energy, we should assign to a hydrogen bond in our model. From the comparison of the monomer radius of gyration, we would conclude that *T* = 0.1853 corresponds to room temperature (300 K), i.e., a temperature *T* in reduced units corresponds to *T*′ = 1619 *T* in Kelvin. From the radius of gyration of the dimer, we would conclude that *T* = 0.172 corresponds to room temperature, leading to *T*′ = 1744 *T* as the conversion rule between reduced and physical temperature units. The difference defines the uncertainty with which we can make the temperature conversion. The average assigns *T* = 0.179 to correspond to room temperature and we obtain a conversion factor of 1676 ± 70. The resulting phase transition temperatures in Kelvin are listed in Table 4. We can also conclude that the energy per hydrogen bond in our model is equal to ε_HB_ = 3.3 ± 0.2 kcal/mol.

**Figure 5:**
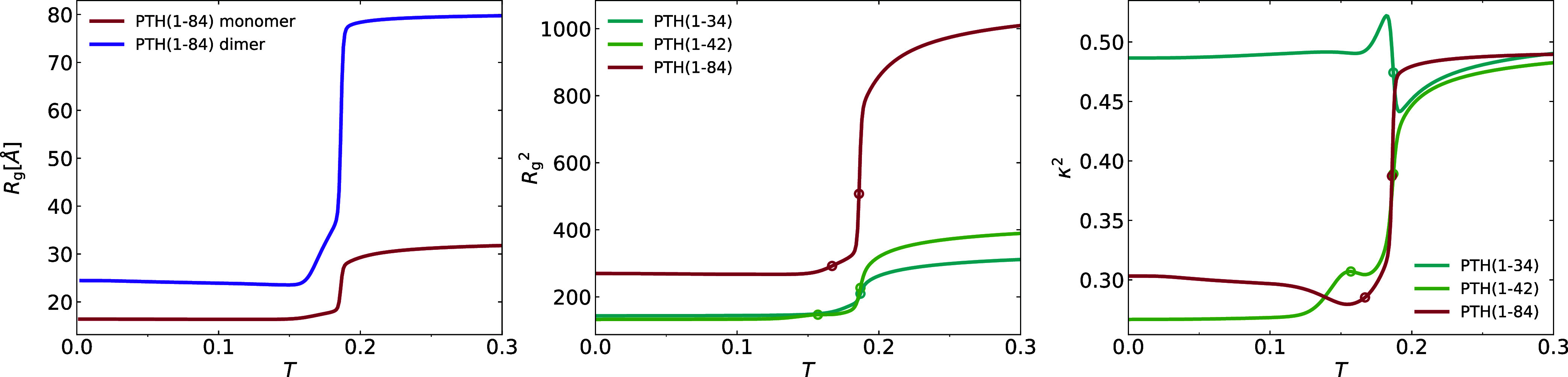
Left plot shows the radius of gyration *R*_g_ of the monomer and the dimer of the PTH_84_ as a function of temperature. The middle plot compares the temperature dependence of the squared radius of gyration *R*_g_^2^ of monomers of the three chain lengths and the right plot shows the shape parameter κ^2^.

**Table 4:** Phase Transition Temperatures T* in Kelvin

system	*T*_low_^*^	*T*_high_^*^
PTH_34_		313 ± 13
PTH_42_	263 ± 11	313 ± 13
PTH_82_	280 ± 12	313 ± 13

The middle part of Figure 5 shows that for all three chain lengths, the radius of gyration of the chains collapses at the position of the high-temperature specific heat peak, in agreement with the findings in refs 14,15 . The shape of the gyration ellipsoid shows no significant change for PTH_34_, but a clear tendency to more spherical structures for the longer chains. One can speculate, especially for the native chain, that the nonaggregating parts fold over the aggregated N-terminal part.

To clarify the secondary structure formation within the chains and the hydrogen bonding pattern underlying the dimerization, we use hydrogen bond matrices, which give the probability for a hydrogen bond between selected backbone repeat units being formed intra- or intermolecularly at a certain temperature. We show these in Figure 6 for *T* = 0.178 = 298 K and in Figure 7 for *T* = 0.15 = 263 K and all three chain lengths in both cases.

**Figure 6:**
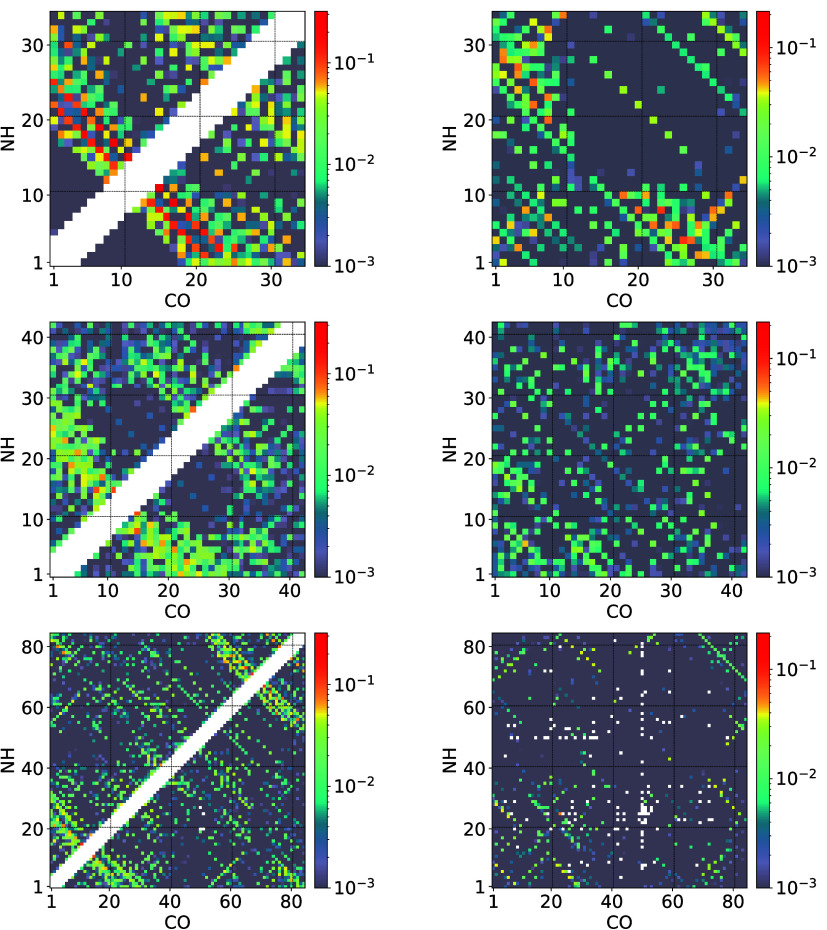
On the left side, we show the intramolecular and on the right side the intermolecular hydrogen bonding pattern at a temperature of *T* = 0.178. The top row is for PTH_34_, the middle row for PTH_42_, and the bottom row for PTH_84_. The color bars on the right give the probability for a hydrogen bond to be formed on a logarithmic scale.

**Figure 7:**
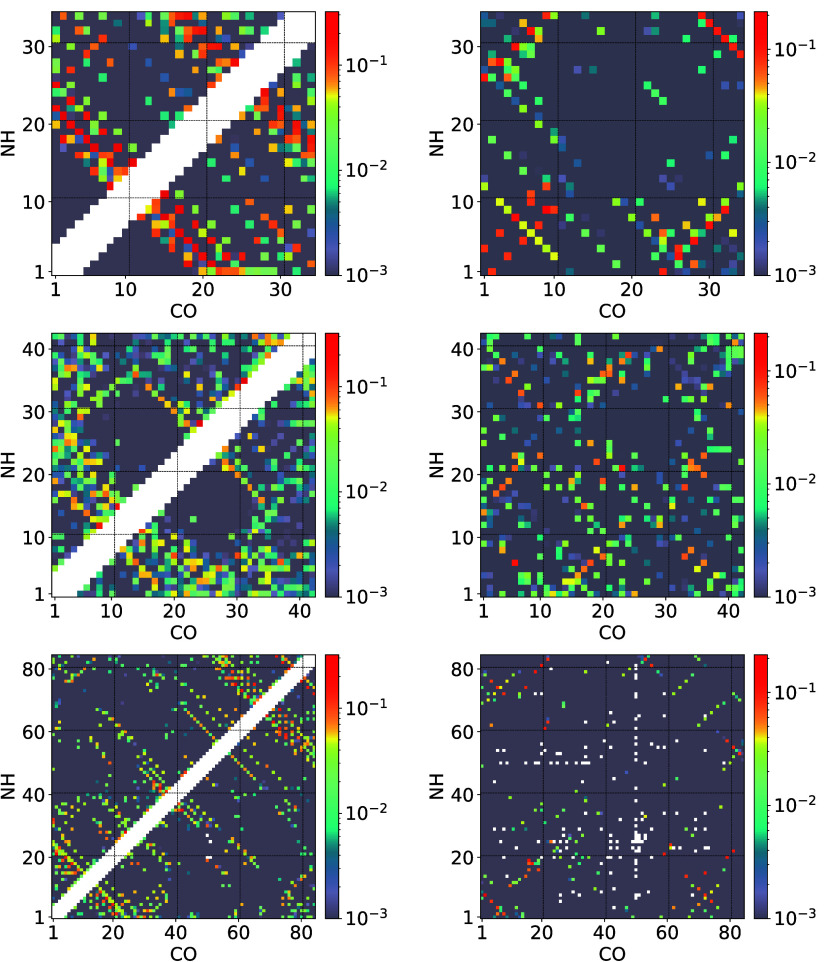
On the left side, we show the intramolecular, and on the right side, the intermolecular hydrogen bonding pattern at a temperature of *T* = 0.15. The top row is for PTH_34_, the middle row for PTH_42_, and the bottom row for PTH_84_. The color bars on the right give the probability for a hydrogen bond to be formed on a logarithmic scale.

The first transition visible in the specific heats clearly leads to a dimer structure with clear intramolecular β-sheet formation, a hydrogen bonding pattern perpendicular to the main diagonal in the plot of Figure 6. This is most pronounced for PTH_34_ where two β-sheet structures are separated by a loop region aground the residues 17–21. This is qualitatively similar to the two α-helices with a linker formed for the same regions in the monomer. Only that in the dimer, the secondary structure is a β-sheet to allow for intermolecular aggregation and hydrogen bond formation visible in the right column. PTH_42_ shows the same behavior with some additional intramolecular hydrogen bonding occurring within the residues 37–39. For PTH_84_, there is a further intramolecular hydrogen bonding pattern developing for the residues 70–84. These additional patterns for PTH_42_ and PTH_84_, however, are all not indicative of consecutive β-sheets forming but more of individual hydrogen bonds occurring for one or the other residue in these ranges. For the two longer chains, it is clearly visible that there is no additional intermolecular hydrogen bonding outside of the N-terminal segment, so it is only this segment which is driving the fibril formation, as we already concluded from the thermodynamic information.

At the lower temperature shown if Figure 7, which is slightly below the low-temperature peaks in the specific heat curves for the two longer chains, the pattern for PTH_34_ has not changed, but the hydrogen bond formation has become more probable, as one would expect at lower temperatures. For PTH_42_, the intramolecular bonding pattern has become better defined and a clearer intermolecular bonding pattern has developed. For PTH_84_, the additional β-sheet like structure at the C-terminus has become well-defined; however, no clear additional intermolecular bonding pattern is developing. We, therefore, conclude that the low-temperature peak in the specific heat curves for the longer chains is mainly due to intramolecular secondary structure formation.

In Figure 8, we show relevant configurations of the aggregated and folded states of dimers for the three chain lengths. However, we want to note that all dimers display significant polymorphism. The right column is for temperatures just below the aggregation transition, and the left column at a temperature below the second transition visible in the specific heat of the two longer segments. These snapshots illustrate the hydrogen bonding patterns discussed in Figures 6 and 7.

**Figure 8:**
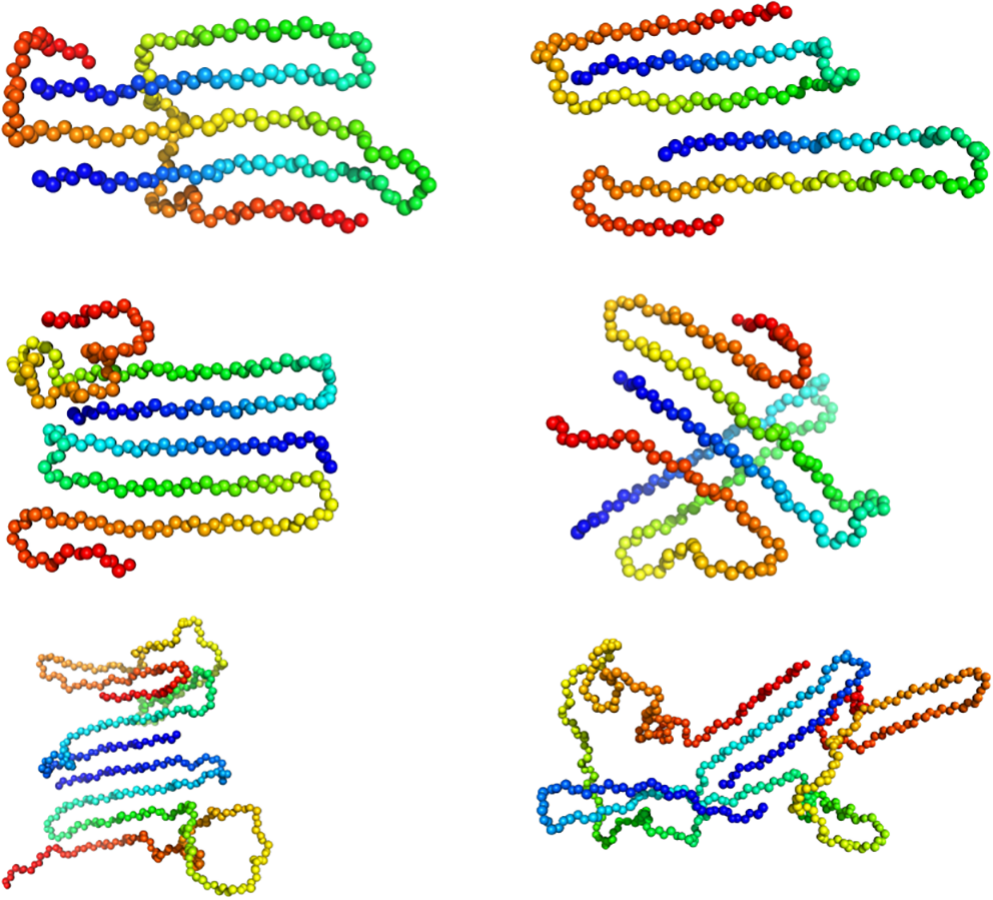
This figure shows relevant configurations of PTH_34_ (top row), PTH_42_ (middle row), and PTH_84_ (bottom row). The left column is for temperatures below *T*_low_^*can^, the right for temperatures between *T*_low_^*can^ and *T*_high_^*can^. The repeat units of the chains are color coded from red (C-terminus) to blue (N-terminus). Only backbone beads are shown.

## Conclusions

We have shown that the thermodynamics of the aggregation of PTH chains is governed by its N-terminal PTH_34_ segment, so aggregation is not a cooperative phenomenon involving interactions between the complete chains. The dimerization is accompanied by a secondary structure change of the PTH_34_ part, which in the monomer folds into two α-helices joined by a flexible linker, whereas in the dimer, the same parts of the chain fold into β-sheets to allow for the chain aggregation. Because of the noncooperativity of the aggregation, and because both the monomer and the dimer show secondary structure in the N-terminal segment, there can only be a small Gibbs free energy gain per repeat unit during the fibrillar aggregation.

A comparison of chain sizes with experimental data furthermore suggests that the dimerization and folding transition occur only slightly above physiological (or room temperature) conditions (around 40 °C). Therefore, one observes a chemical equilibrium between monomer and dimer with measurable concentration of both^4^ in room temperature in vitro experiments at low enough concentrations. There is only a small Gibbs free energy gain with each chain added to the aggregate until, finally, a mature fibril nucleates. These considerations also mean that the addition of a single chain to an aggregate is a reversible process under physiological conditions, which is a prerequisite for the functional use of the fibril.
